# Simultaneous monitoring of the drug release and antitumor effect of a novel drug delivery system-MWCNTs/DOX/TC

**DOI:** 10.1080/10717544.2016.1233592

**Published:** 2017-02-03

**Authors:** Xia Dong, Zhiting Sun, Xiaoxiao Wang, Dunwan Zhu, Lanxia Liu, Xigang Leng

**Affiliations:** Tianjin Key Laboratory of Biomedical Materials, Institute of Biomedical Engineering, Chinese Academy of Medical Sciences and Peking Union Medical College, Tianjin, PR China

**Keywords:** Drug delivery, noninvasive imaging, tumor therapy, carbon nanotubes, DOX

## Abstract

Monitoring drug release and therapeutic efficacy is crucial for developing drug delivery systems. Our preliminary study demonstrated that, as compared with pristine multiwalled carbon nanotubes (MWCNTs), transactivator of transcription (TAT)-chitosan functionalized MWCNTs (MWCNTs-TC) were a more promising candidate for drug delivery in cancer therapy. In the present study, a MWCNTs/TC-based drug delivery system was developed for an anticancer drug, doxorubicin (DOX). The drug loading and *in vitro* release profiles, cellular uptake and cytotoxicity were assessed. More importantly, the *in vivo* drug release and antitumor effect of MWCNTs/DOX/TC were evaluated by noninvasive fluorescence and bioluminescence imaging. It was demonstrated that MWCNTs/DOX/TC can be efficiently taken up by BEL-7402 hepatoma cells. The release of DOX from MWCNTs/DOX/TC was faster under lower pH condition, which was beneficial for intrcellular drug release. The *in vivo* release process of DOX and antitumor effect in animal model were monitored simultaneously by noninvasive fluorescence and luminescence imaging, which demonstrated the application potential of MWCNTs/DOX/TC for cancer therapy.

## Introduction

Multiwalled carbon nanotubes (MWCNTs) are seamless hollow tubes formed by rolling of multiple layers of graphene, which have recently gained popularity as potential delivery vehicles for therapeutic/diagnostic agents due to their unique structural, optical, electronic and chemical properties (Qin et al., 2011a; Sajid et al., 2016). Owing to their high surface area ratio, MWCNTs have strong loading capacity for a variety of agents including biomolecules (proteins or nucleic acids) and chemical drugs (either hydrophilic or hydrophobic) (Li et al., 2014; Qi et al., 2015). They can also increase the bioavailability of these molecules by controlling their release or protecting them from degradation or alleviate their toxicity and harmful side effects through targeted delivery (Mu et al., 2009; Li et al., 2016; Mehra & Palakurthi, 2016). These features make it possible to develop multifunctional drug carriers to further improve the efficacy of therapeutic/diagnostic agents.

The main problem limiting the medical application of MWCNTs is their lack of solubility and dispersivity in aqueous media, almost in any kind of solvent (Peretz, 2012). To overcome the restriction of their inherent hydrophobicity, functionalization of MWCNTs by either covalent or noncovalent approach was widely utilized to improve their solubility and lower their toxicity in biological systems (Sitko, 2012; He et al., 2013). As compared to covalent functionalization, noncovalent approach has some advantages like easy handling and nondestructive of the graphene lattice. A wide variety of molecules can be attached to the basic structure of CNTs via van der Waals force, π-stacking interactions and electrostatic force (Wong et al., 2013), which include nucleic acids (Das et al., 2011; Wu et al., 2011), peptides (Liu et al., 2007; Iancu et al., 2011; Hashida et al., 2014), polymers (Nabid et al., 2012; Karadas & Ozkan, 2014) and surfactants (Duch et al., 2011; Wang et al., 2012).

Chitosan (CS), a cationic polysaccharide obtained from deacetylation of chitin, has attracted intense attention for application as a drug/gene delivery vehicle (Misra et al., 2012) or tissue engineering scaffold (Depan, 2014) because of its hydrophilicity, biocompatibility, biodegradability and low immunogenicity properties. TAT (transactivator of transcription) peptide, a widely recognized cell-penetrating peptide, can drive macromolecules to penetrate the cell membrane and target the cell nucleus (Liu et al., 2008; Qin et al., 2011b; Mei et al., 2014), while causing no damage to the cell membrane. Our preliminary study demonstrated that, as compared with the pristine MWCNTs, TAT-chitosan conjugate (TC) functionalized MWCNTs (MWCNTs-TC) were more water soluble with much lower cytotoxicity, higher cellular uptake and enhanced accumulation in tumor tissues (Dong et al., 2015), making them promising candidates for drug delivery vehicles in cancer therapy.

Monitoring of drug release and therapeutic efficacy is crucial for developing drug delivery systems. With the rapid development of biomedical imaging technology, real-time tracking of drug release *in vivo* has become a major challenge. The usual evaluation methods for drug release include *in vitro, ex vivo* and *in vivo* assays, such as high-performance liquid chromatography (HPLC), radio-labeling and detection, histological and immunohistochemical assays. However, the conventional evaluation methods might not provide accurate information in a real-time manner without radiation, because the test samples must be collected and evaluated *ex vivo*. Moreover, due to the different condition between *ex vivo* and *in vivo* situation, *ex vivo* evaluation assays may not fully reflect the *in vivo* behavior of the drug delivery system (Wohl-Bruhn et al., 2012). Therefore, *in vivo* imaging techniques should be more valuable because they can track and evaluate the system with features of real-time, nondestructive, longitudinal and quantitative analysis (Zhou et al., 2015). Compared to other imaging patterns, optical imaging presents a more promising prospect because of its significant benefits in safe and precise examination. The continuous efforts and development in device make optical imaging such as fluorescence imaging and bioluminescence imaging as advanced tools for *in vivo* tracking or diagnosis. Mi Kyung Kwak et al. have successfully applied bioluminescence imaging to evaluate the suppressive effects on *in vivo* tumor growth of a thermosensitive hydrogel containing antitumor drug (Kwak et al., 2010). In addition, fluorescence imaging technology has also been used to monitor and track the biomaterial metabolism and drug delivery *in vivo* (Hoffmann et al., 2012; Dong et al., 2016). These studies indicated that *in vivo* optical imaging is more sensitive and could be a valuable noninvasive monitoring tool for the assessment of drug release and dynamics of tumor growth.

In the present study, MWCNTs-TC were used as the delivery vehicle of antitumor drug doxorubicin hydrochloride (DOX) to assess its application effect in cancer therapy. More importantly, the *in vivo* drug release and antitumor effect of MWCNTs/DOX/TC were simultaneously monitored by fluorescence and bioluminescence imaging assays, which directly demonstrated the sustained release and therapeutic effect of the drug delivery system and revealed the application potential of MWCNTs/DOX/TC for cancer therapy.

## Materials

MWCNTs-COOH (purity of 98% with -COOH content of 3.86%, 0.5–2 μm in length with a diameter of less than 8 nm) were purchased from Chengdu Organic Chemicals Co., Ltd. (Sichuan, People’s Republic of China). Chitosan (molecular weight of 5000–8000 Da and degree of deacetylation of 90%) was obtained from Golden-Shell Biochemical Co., Ltd. (Yuhuan, Zhejiang, People’s Republic of China). DOX hydrochloride was purchased from Dakub Meilun Biology Technology Co., Ltd. (Dalian, People’s Republic of China). Fetal bovine serum (FBS) was provided by Gibco® (Thermo Fisher Scientific, Waltham, MA). TAT peptide with the amino acid sequence of YGRKKRRQRRR was synthesized by SBS Genetech Co., Ltd. (Beijing, People’s Republic of China). D-Luciferin was purchased from Gold Biotechnology, Inc. (St. Louis, MO)

### Preparation and characterization of MWCNTs/DOX/TC

TC conjugates were synthesized according to the method described in our previous publication (Dong et al., 2015). To prepare MWCNTs/DOX/TC, DOX at varied concentrations was mixed with MWCNTs-COOH dispersed in phosphate-buffered saline (PBS) solution and stirred overnight at room temperature in dark conditions. After that, TC was added to the mixture with mass ratio of TC to MWCNTs being 4:1 and stirred for 5–6 h to form the MWCNTs/DOX/TC complexes. Unloaded DOX was removed by dialyzing the reaction mixture against PBS until the dialysate became colorless. Encapsulation efficiency and drug-loading capacity of DOX onto MWCNTs were quantified at 480 nm by UV-vis spectroscopy based on a standard curve of DOX. Encapsulation efficiency and drug-loading capacity were calculated according to the following equations:

Drug-loading efficiency (%) = (weight of loaded drug)/(weight of feeding drug) × 100%

Drug-loading capacity (%) = (weight of loaded drug)/(weight of loaded drug + weight of MWCNTs) × 100%

MWCNTs/DOX/TC were characterized with an infrared spectroscope (Nicolet 2000, Thermo Fisher Scientific, Waltham, MA) from 4000 to 400 nm^−^ ^1^ on KBr plates. UV-Visible (UV-Vis) absorption spectra were recorded using a microplate reader (The Varioskan™ Flash, Thermo Fisher Scientific). The surface morphology of MWCNTs-COOH, MWCNTs/DOX and MWCNTs/DOX/TC were analyzed by transmission electron microscopy (JEM-1010; JEOL, Tokyo, Japan). The zeta potential was measured with a Zetasizer Nano-ZS instrument (Malvern Instruments, Malvern, UK).

### *In vitro* DOX release study

To investigate the *in vitro* release profile of MWCNTs/DOX/TC and MWCNTs/DOX, 2 mL samples were added into a dialysis bag (*M*_w_ cutoff =3.5 KDa) and dialyzed against 100 mL PBS under two different pH values (7.4 or 5.5) with stirring at 37 °C. At the predetermined time points, 3 mL dialysate was collected to measure DOX absorbance at 480 nm, and the same volume of fresh PBS was added to keep the volume of dialysate constant. The concentration of DOX was quantitatively calculated by referring to a standard curve of DOX.

### Cytotoxicity assessment

The effect of free DOX or MWCNTs/DOX/TC on human liver cancer cells BEL-7402 was evaluated with Cell Counting Kit-8 (Dojindo Molecular Technologies, Inc., Kumamoto, Japan) according to the manufacturer’s instructions. Briefly, the cells were seeded in 96-well plates at 10^4^ cells per well in 100 μL of RPMI 1640 medium (HyClone Laboratories, Inc., Logan, UT) and grown at 37 °C in a 5% CO_2_ atmosphere for 24 h, and then MWCNTs/DOX/TC, MWCNTs/TC or free DOX of varied concentrations was added to incubate with the cells for 24, 48 and 72 h, respectively. After that, 10 μL CCK-8 solution was added and the optical absorbance at 450 nm was measured using a microplate reader (The Varioskan™ Flash; Thermo Fisher Scientific, Waltham, MA).

### Cellular uptake

Briefly, BEL-7402 cells were seeded into 96-well plates at the density of 10^4^ cells per well and allowed to grow overnight and subsequently incubated with different concentrations of MWCNTs/DOX/TC or free DOX for 4 h and 12 h, the cells were then washed three times with PBS and fixed with immune staining fix solution for 20 min at room temperature, followed by the labeling of the intracellular microfilament with actin-tracker green and subsequent staining of the nucleus with DAPI (Beyotime Institute of Biotechnology, Shanghai, China). The cellular uptake of MWCNTs/TC/DOX or DOX was analyzed with the GE IN Cell Analyzer 2000 High-Content Cellular Analysis System (GE Healthcare Bio-Sciences Corp., Piscataway, NJ)

### *In vivo* monitoring of DOX release and antitumor efficiency

Balb/c nude mice (6 weeks, male) were purchased from Peking Union Medical College. All animal procedures were conducted following the protocol approved by the Institutional Laboratory Animal Ethics Committee, and all animal experiments were performed in compliance with the Guiding Principles for the Care and Use of Laboratory Animals, Peking Union Medical College. Luciferase-expressing Bel-7402 cells (1 × 10^6^) in 0.1 mL normal saline (NS) were injected into the armpit region of Balb/c nude mice. When the volume of tumors reached to ∼100 mm^3^, the mice were divided into different treatment groups (6 mice/group) and free DOX or MWCNTs/DOX/TC at the dose of 10 mg/kg, 20 mg/kg or 30 mg/kg DOX were injected into the center of tumor with NS-treated mice as the negative control. Fluorescence imaging was conducted to monitor the drug release process using an live animal imaging system (IVIS Lumina system, Xenogen, Alameda, CA) until the fluorescence signal disappeared, with excitation wavelength being set at 532 nm. To investigate the antitumor effect, luminescence imaging was conducted 5 min after intraperitoneal injection of 200 μL luciferin (15 mg/mL) using the IVIS Lumina imaging system for a period of 25 days.

### Statistical analysis

Data were presented as the mean of six individual observations with the standard deviation. The statistical analysis was performed using the Bonferroni *t*-test. Statistical significance was determined at *p *<* *0.05.

## Results and discussion

### Characterization of MWCNTs/DOX/TC

Our preliminary study demonstrated MWCNTs/TC were more water soluble with much lower cytotoxicity, higher cellular uptake and enhanced accumulation in tumor tissues compared with the pristine MWCNTs. Therefore, a MWCNTs/TC-based drug delivery system for DOX was developed through noncovalent approach, and its drug delivery profile and antitumor effect were investigated in the present study.

**Figure 1.**
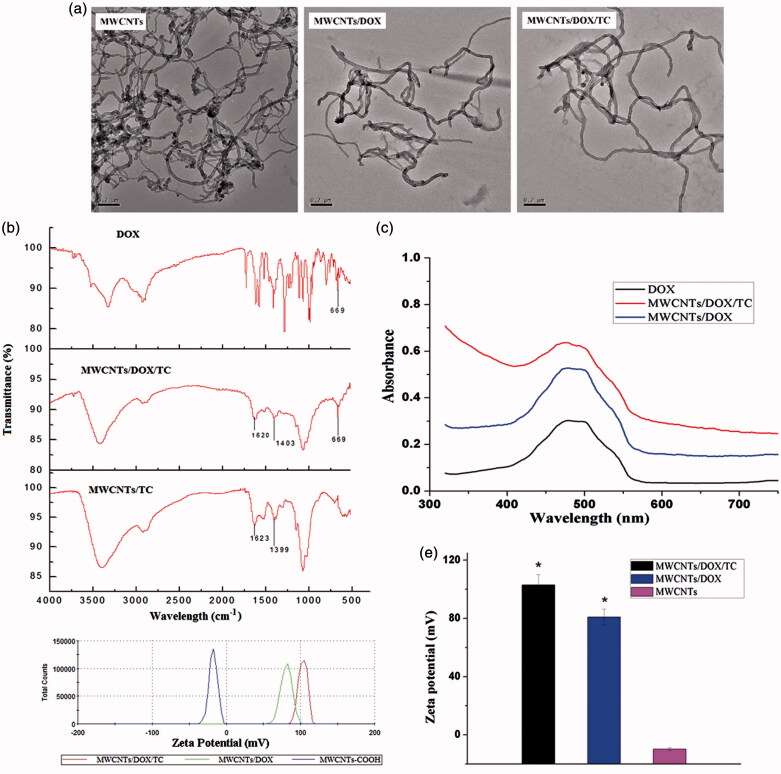
Characterization of MWCNTs/DOX/TC. (a) Transmission electron microscopic images; (b) FTIR spectra; (c) UV-vis absorption spectra; (d and e) Zeta potential of MWCNTs, MWCNTs/DOX and MWCNTs/DOX/TC.

First, the MWCNTs/DOX/TC was characterized by transmission electron microscope, Fourier transform infrared spectroscopy and UV-Vis absorption spectroscopy. Transmission electron microscope was utilized to observe the morphology MWCNTs-COOH, MWCNTs/DOX and MWCNTs/DOX/TC. Figure 1(a) showed that no detectable morphological difference was found among them and the dispersibility of MWCNTs/DOX/TC was improved. Infrared spectroscopic analysis revealed the existence of the characteristic peaks of benzene ring of DOX at 669 cm^−^^1^ on MWCNTs/DOX/TC (Figure 1b). UV absorption spectroscopic analysis showed the main absorption of DOX at 490 nm for MWCNTs/DOX/TC without red-shifted (Figure 1c). All these data suggested the successful loading of DOX on MWCNTs by physical adhesion.

Zeta potential is one of the key factors determining the application potential of a drug delivery system. The data shown (Figure 1(d,e)) illustrated that the average zeta potential was 103 ± 7.32 mV for MWCNTs/DOX/TC and 80.9 ± 3.35 mV for MWCNTs/DOX, which were much higher than that for MWCNTs-COOH (−11.6 ± 2.43 mV). MWCNTs-COOH have a negative potential because of the COO^-^ groups on the end or sidewall of MWCNTs; however, both TC and DOX contain a large number of cationic groups, which led to the elevated zeta potential of MWCNTs/DOX or MWCNTs/DOX/TC.

### Drug-loading and release studies *in vitro*

Drug-loading and release behavior is one of the most important factors determining the application potential of drug delivery systems. Previous reports have shown that DOX can be absorbed onto the surface of modified SWCNTs via π–π interactions (Liu et al., 2007). It was also reported that the DOX-loading efficiency was influenced by the zeta potentials of the drug carrier (Zhang et al., 2009), illustrating that the electrostatic interactions played an important role in the absorption process. Our preliminary study found that modification with positively charged TC molecules significantly attenuated the DOX-loading capacity of MWCNTs (data not shown). Therefore, to achieve higher DOX-loading capacity, DOX was first loaded onto the surface of MWCNTs, followed by further coating of the MWCNTs/DOX complexes with TC for higher cellular uptake of the drug delivery system.

**Figure 2.**
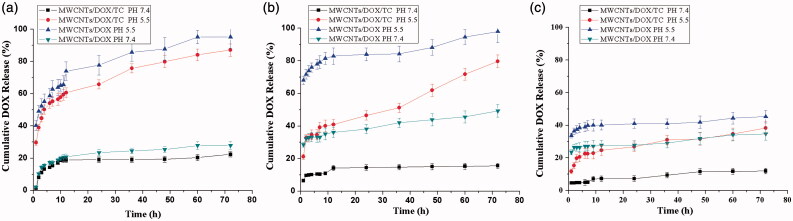
Drug release from MWCNTs/DOX/TC and MWCNTs/DOX. (a) DOX/MWCNTs = 3/1; (b) DOX/MWCNTs = 1/1; (c) DOX/MWCNTs = 1/2.

The drug-loading capacity and efficiency of the MWCNTs/DOX/TC with different DOX to MWCNTs mass ratios (3/1, 1/1 and 1/2, respectively) were further investigated. The data revealed that the DOX-loading capacity was improved with increasing DOX/MWCNT ratio (about 24%, 33% and 50% for mass ratio of 1/2, 1/1 and 3/1, respectively), whereas the loading efficiency demonstrated the opposite trend (about 63%, 50% and 35% for mass ratio of 1/2, 1/1 and 3/1, respectively). As shown in Figure 2, that the DOX release rate was increased with increasing DOX/MWCNTs ratio in PBS at pH of both 7.4 and 5.5. It was also found that DOX was released at a significantly lower rate at pH 7.4 from MWCNTs/DOX/TC than that at pH 5.5, which is beneficial for intracellular drug delivery and release. Coating of MWCNT/DOX with TC significantly slowed the release efficiency of DOX from MWCNTs/DOX/TC by comparing with MWCNTs/DOX at each condition.

### Cellular uptake observation

**Figure 3.**
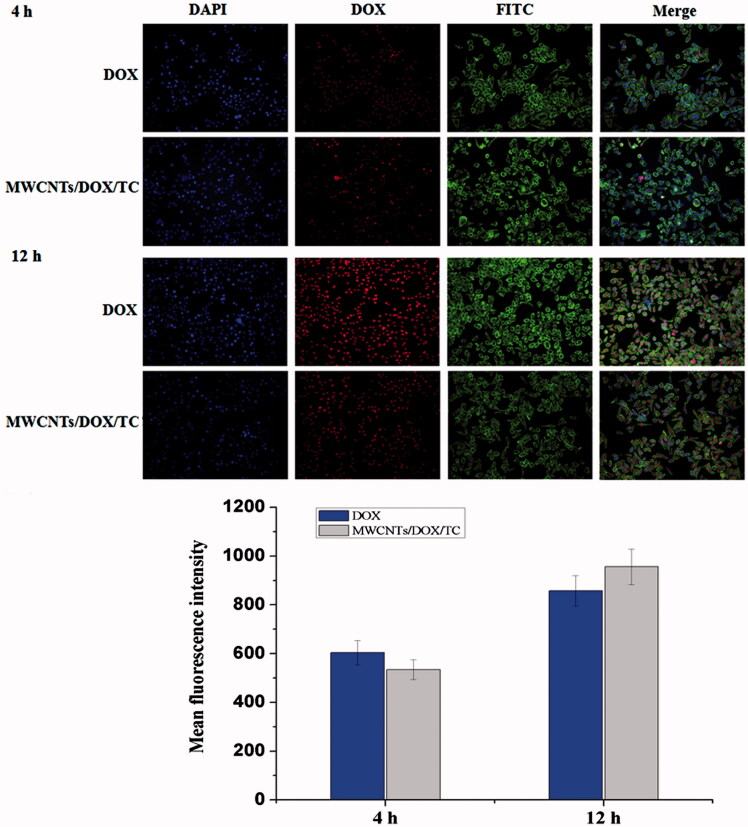
Cellular uptake of BEL-7402 treated with free DOX or MWCNTs/DOX/TC for 4  h and 12 h. Notes: Cell nucleus (Blue, DAPI); DOX (Red); Microfilament (Green, FITC).

Cellular uptake of MWCNTs/DOX/TC or free DOX was analyzed with IN Cell Analyzer 2000. Figure 3 showed the intracellular fluorescence intensity of DOX after incubating BEL-7402 cells with MWCNTs/DOX/TC or free DOX for 4 or 12 h. The intracellular fluorescence intensity of DOX after incubation for 12 h was stronger than that for 4 h, which indicated that MWCNTs/DOX/TC could efficiently enter the tumor cells. Quantitative analysis further demonstrated that the mean florescence intensity of free DOX group is slightly higher than that of MWCNTs/DOX/TC group with equivalent concentration of DOX after incubation for 4 h, which might be due to the fast cellular internalization of DOX through the cell membrane by a passive diffusion mechanism (Prabaharan et al., 2009). However, after incubation for 12 h, the florescence intensity of DOX for MWCNTs/DOX/TC group was higher, which indicated that more DOX was still retained in the tumor cells due to the prolonged DOX release from the drug delivery system. More importantly, most of the nucleus of the MWCNTs/DOX/TC treated cells was filled with DOX, indicating the successful release of DOX from the delivery system, as our previous study demonstrated that MWCNTs/TC could efficiently enter the tumor cells and mainly accumulated in the cytoplasm, but cannot cross nuclear membranes. All these results implied that MWCNTs/DOX/TC led to more efficient DOX delivery and release in tumor cells.

### Assessment of *in vitro* antitumor activity

**Figure 4.**
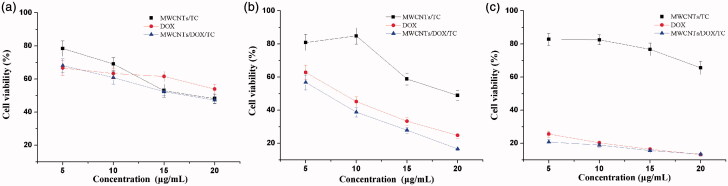
Cytotoxicity of MWCNTs/DOX/TC at 24 h (a), 48 h (b) and 72 h (c).

The cytotoxicity of MWCNTs/DOX/TC against BEL-7402 cells was assessed and compared with that of MWCNTs/TC and free DOX. The results shown in Figure 4 illustrated that the viability of MWCNTs/DOX/TC treated cells decreased in a dose-dependent manner, with exposure to MWCNTs/DOX/TC at 20 μg/mL leading to a ∼45% viability decrease at the initial 24-h postincubation. However, as compared with free DOX, MWCNTs/DOX/TC induced slightly higher cytotoxicity after incubation for 24 and 48 h, and no significant difference was found between MWCNTs/DOX/TC and DOX group at each concentration for 72 h of exposure. It was also observed that the viability of MWCNTs/TC treated cells was decreased slightly with increasing concentration for 24 and 48 h; however, the cell viability bounced back and gradually recovered at 72 hours after incubation. According to the data from cellular uptake study and cytotoxicity assessment, we speculated that the higher cytotoxicity of MWCNTs/DOX/TC was the result of the combined action of DOX and MWCNTs/TC.

### Simultaneous monitoring of drug release and the antitumor effect of MWCNTs/DOX/TC by noninvasive imaging

Although CNTs has been reported to be an efficient carrier for controlled release of antitumor drug DOX (Ali-Boucetta et al., 2008; Liu et al., 2009), little information has been accumulated regarding the *in vivo* release of DOX from the CNTs/DOX system due to the lack of accurate quantification technology suitable for live animal. Compared to other conventional monitoring methods like tissue resection which entails animal sacrifice, noninvasive *in vivo* imaging technology is more intuitive and visible (Hoffmann et al., 2012; Appel et al., 2013). Current *in vivo* imaging modalities include ultrasonography (US), computed tomography (CT), single-photon emission computed tomography(SPECT) and magnetic resonance imaging (MRI) (Othman et al., 2012; Fischerauer et al., 2013), etc. Although these imaging patterns are very valuable for *in vivo* evaluation, optical imaging has been attracting increasing attention as an advanced tool for tracking the *in vivo* fate of biomaterials, drugs and cells, because of its numerous advantages including easy setup, low cost, high sensitivity, low-energy radiation, noninvasion and long-term observation. In the present study, fluorescence imaging was employed for monitoring the *in vivo* release process of DOX from the MWCNTs/DOX/TC delivery system, taking advantage of the unique fluorescence property of DOX. In the meantime, the location of tumor and the change of tumor volume could be determined precisely by assessing the radiance efficiency of luciferase-expressing BEL7402 cells through bioluminescence imaging.

**Figure 5.**
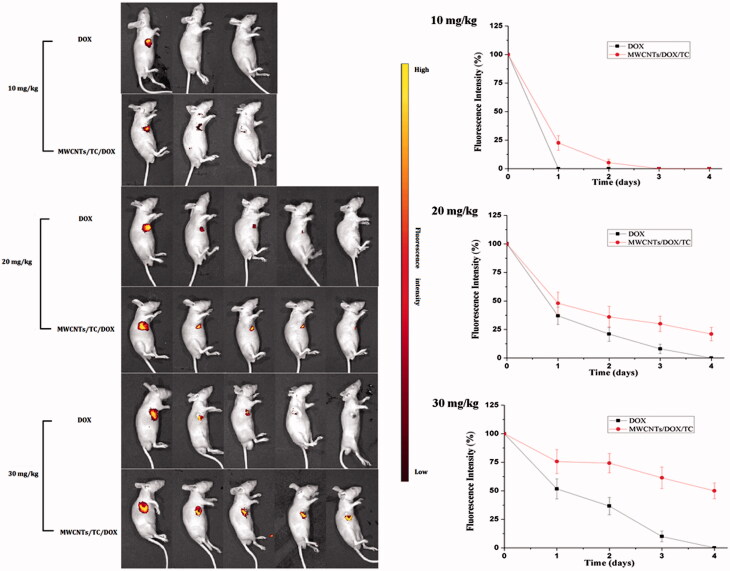
Fluorescence imaging for *in vivo* tracking of drug release and quantitative analysis of fluorescence.

To investigate the *in vivo* drug release efficiency and anti-tumor effect of MWCNT/DOX/TC more accurately, different concentrations of MWCNTs/DOX/TC or free DOX were injected into the center of tumor, and fluorescence imaging and bioluminescence imaging were simultaneously conducted for the evaluation of the delivery system. The change in fluorescence or bioluminescence intensity can be monitored noninvasively in a real-time manner to reflect the drug release rate and inhibition of tumor growth simultaneously. As shown in Figure 5, the release of DOX can be tracked by fluorescence imaging. By quantitative analysis of the total fluorescence intensity in the efficiency region of tumor tissues, the relative release of DOX can be monitored drastically. It was revealed by the fluorescence imaging that a slower release rate was achieved for MWCNTs/DOX/TC group, and MWCNTs/DOX/TC significantly elongated the retention time of DOX in the tumor tissues at each concentration (10 mg/kg, 20 mg/kg or 30 mg/kg of DOX) as compared with free DOX. No fluorescence signal was observed one  day after injection of 10 μg/mL free DOX, whereas the fluorescence remained for more than 24 h and a faint fluorescence signal could still be detected after 2 days in the MWCNTs/DOX/TC group with equivalent amount of DOX. Similarly, at the concentration of 20 μg/mL, ∼50% fluorescent signal remained in the tumors in MWCNTs/DOX/TC group versus a 35% remanence in the free DOX group on the first day. The fluorescence signals in the free DOX group disappeared completely 4 days after injection, while around 20% of the fluorescence intensity still remains at day 4 in the MWCNTs/DOX/TC group. When DOX was at 30 μg/mL, approximately 60% of the fluorescence intensity still remained in the tumors at day 4 for the MWCNTs/DOX/TC group, while the fluorescence in the tumors disappeared completely for the free DOX group. All these results indicated that MWCNTs/DOX/TC realized a conspicuously sustained release of DOX, and the sustained release of DOX could be extended by increasing DOX concentration. More importantly, it was also demonstrated that the difference in the release process of different DOX concentration group could be intuitively and clearly observed by fluorescence imaging.

**Figure 6.**
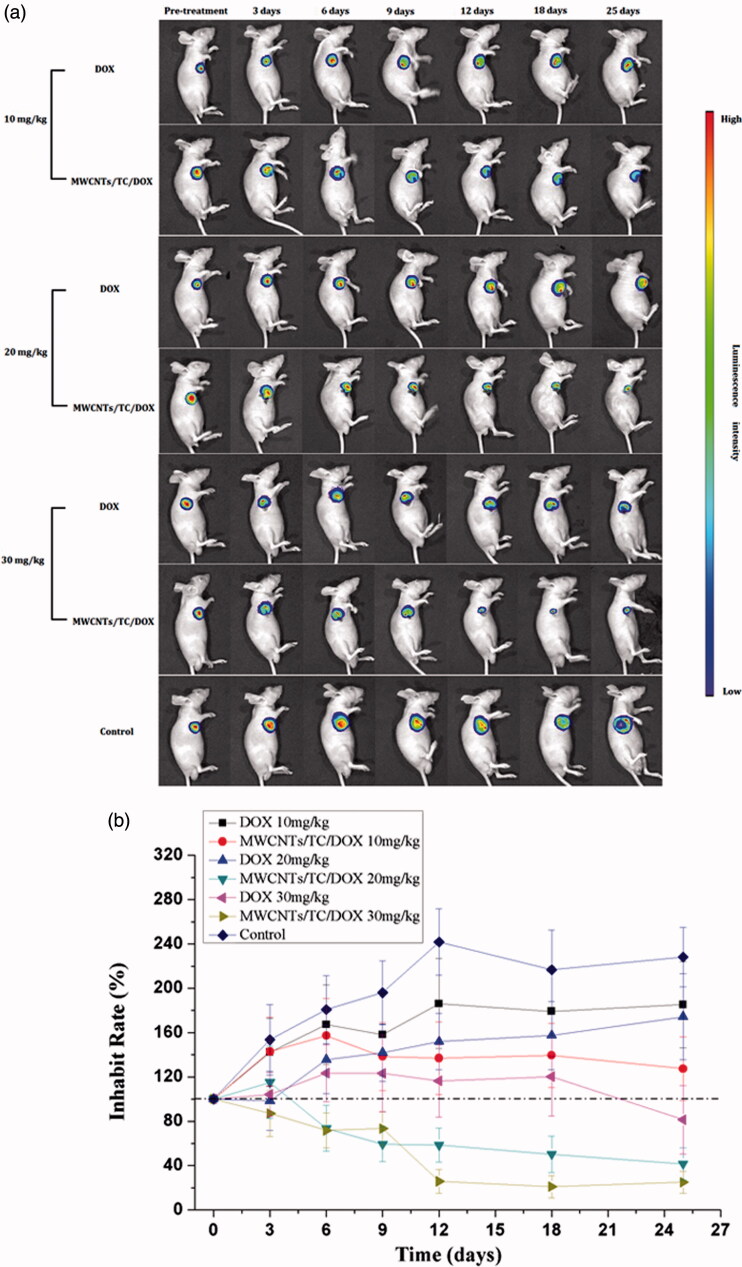
Bioluminescence imaging for monitoring antitumor effect. (a) Bioluminescence imaging. (b) Quantitative analysis of luminescence intensity.

Noninvasive bioluminescence imaging is a powerful imaging method and able to detect 400 to 1000 cancer cells inoculated subcutaneously or intraperitoneally and 1000 to 10 000 cancer cells inoculated intravenously, which is far more sensitive than any of the other noninvasive imaging techniques (Klerk et al., 2007). Due to its spectral characteristics and convenience to perform, this technique is widely used to track cancer metastasis and the effects of antineoplastic therapy in animal models (Kim et al., 2015). In the present study, the *in vivo* antitumor efficacy of MWCNTs/DOX/TC was investigated by assessing the radiance efficiency of luciferase-expressing BEL7402 cells with the change of tumor volume. Over a period of 25 days observation following treatment with different concentrations of MWCNTs/DOX/TC or free DOX, tumor inhibition was assessed by noninvasive bioluminescence imaging. The antitumor effect of MWCNTs/DOX/TC was obviously observed in Figure 6(a). Further quantitative analysis of luminescence intensity (Figure 6b) revealed a significant difference in tumor volume between MWCNTs/DOX/TC and free DOX treatment group at each DOX concentration. MWCNTs/DOX/TC with 10 mg/kg DOX did not induce any tumor size reduction as compared to the initial tumor size but did demonstrate significantly stronger anti-tumor effect than free DOX at the same concentration. Treatment with MWCNTs/DOX/TC at 20 mg/kg and 30 mg/kg for 12 days resulted in a tumor size reduction of about 40% and 75%, respectively, while the tumor size in the mice treated with free DOX at 20 and 30 mg/kg was not reduced as compared to the initial tumor size, but was about 37% and 52% smaller than that in the control group. Although no significant difference in the *in vitro* cytotoxicity against hepatoma cells was observed between MWCNTs/DOX/TC and free DOX-treated group during the period of 3 days incubation, it was obvious that the *in vivo* therapeutic efficiency of the MWCNTs/DOX/TC was significantly superior over that of free DOX. The discrepancy between the *in vitro* and *in vivo* effect is probably due to the sustained release of DOX from MWCNTs/DOX/TC delivery system *in vivo*, which led to longer-lasting effective drug concentration maintained in the tumor tissues.

## Conclusions

In summary, in this study, an effective anticancer drug delivery vehicle MWCNTs/DOX/TC was developed with high drug loading effectiveness and pH-dependent controlled release. The *in vivo* release process of DOX and antitumor effect were monitored simultaneously by noninvasive fluorescence and luminescence imaging, which demonstrated the application potential of MWCNTs/DOX/TC for cancer therapy.
